# Fat/carbohydrate ratio but not energy density determines snack food intake and activates brain reward areas

**DOI:** 10.1038/srep10041

**Published:** 2015-05-14

**Authors:** Tobias Hoch, Silke Kreitz, Simone Gaffling, Monika Pischetsrieder, Andreas Hess

**Affiliations:** 1 Food Chemistry Unit, Department of Chemistry and Pharmacy, Emil Fischer Center, Friedrich-Alexander Universität Erlangen-Nürnberg (FAU), Erlangen, Germany; 2 Institute of Experimental and Clinical Pharmacology and Toxicology, Emil Fischer Center, Friedrich-Alexander Universität Erlangen-Nürnberg (FAU), Erlangen, Germany; 3 Pattern Recognition Lab, Friedrich-Alexander Universität Erlangen-Nürnberg (FAU), Erlangen, Germany; 4 School of Advanced Optical Technologies (SAOT), Friedrich-Alexander Universität Erlangen-Nürnberg (FAU), Erlangen, Germany

## Abstract

The snack food potato chips induces food intake in ad libitum fed rats, which is associated with modulation of the brain reward system and other circuits. Here, we show that food intake in satiated rats is triggered by an optimal fat/carbohydrate ratio. Like potato chips, an isocaloric fat/carbohydrate mixture influenced whole brain activity pattern of rats, affecting circuits related e.g. to reward/addiction, but the number of modulated areas and the extent of modulation was lower compared to the snack food itself.

Ad libitum availability of palatable food may lead to hedonic hyperphagia, i.e. increased energy intake and, consequently, elevated body weight gain due to a change in food intake behaviour pattern^1^. To trigger food intake beyond satiety, factors must be involved that overrule the homeostatic energy balance and satiety via different signalling pathways of a non-homeostatic reward system^2^. As shown before, the intake of the snack food potato chips strongly modulates the activity within the brain reward system in ad libitum fed rats. Additionally it leads to significantly different activation of brain regions regulating food intake, satiety, sleep, and locomotor activity^3^. Behavioural studies confirmed that energy intake and feeding-related locomotor activity were elevated when potato chips were available^3^. Although the neurobiological regulation of food intake is much more complex than the regulation of drug addiction, some striking overlaps of neurophysiological mechanisms, brain activation pattern, and behavioural consequences have been controversially discussed^7^. The brain circuitry involved is strongly activated by food intake after restriction, but also by the intake of highly palatable foods in particular^0^. In general, highly palatable food is high-caloric and/or rich in fats and/or carbohydrates. Thus, it has been hypothesized that the food’s energy density may be the crucial factor that triggers food intake beyond satiety resulting in elevated weight and, eventually, in obesity^2^.

A recent behavioural study revealed that fats and carbohydrates are the major molecular determinants of the palatability of snack food^3^. Furthermore, the energy content of potato chips is mainly (94%) determined by the fat and carbohydrate content. Therefore, it could be assumed that the energy content is the driving force of hedonic hyperphagia in the case of potato chips. Consequently, we conducted behavioural preference tests to investigate the intake of foods with different fat/carbohydrate contents and performed magnetic resonance imaging (MRI) measurements to investigate the modulation of whole brain activity induced in rats.

## Results and Discussion

**Figure 1:**
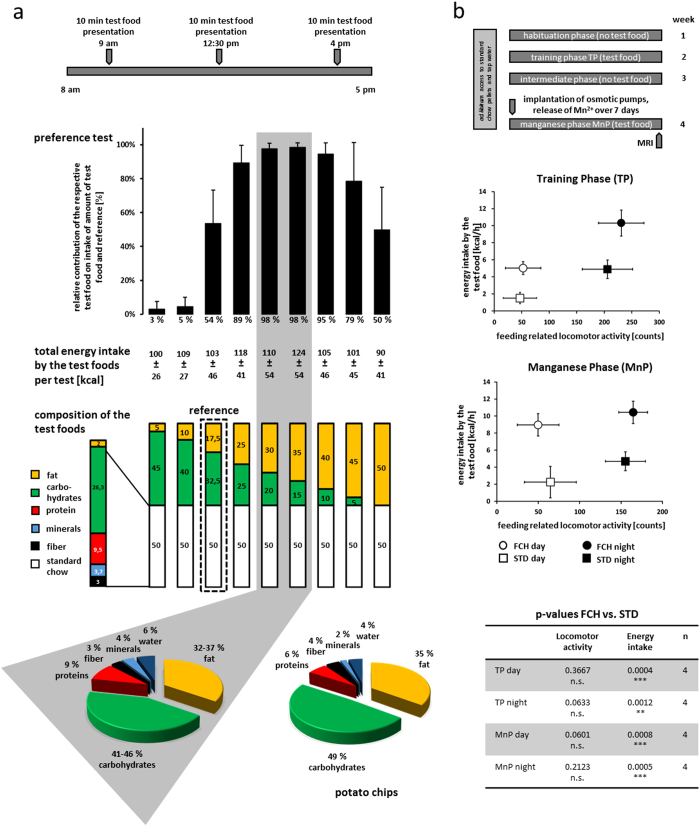
(a) Activity of test foods with different fat/carbohydrate ratios to induce additional food intake during short-term test food presentation (10 minutes) in two-choice preference tests. Differences in energy intake per test food compared to the reference (17.5% fat, 32.5% carbohydrates and 50% STD) are displayed as relative contribution of the respective test food to the total intake of test and reference food (mean ± SD). Below, the composition of test foods is shown and the most attractive mean composition is compared with the composition of potato chips. **(b) Energy intake and respective feeding-related locomotor activity during phases of 7 days continuous test food presentation.** Both factors are shown in their dependency on the test foods [standard chow (STD) or a mixture of 35% fat and 65% carbohydrates (FCH)] in the training phase (TP) and the manganese phase (MnP) during the 12/12 h light/dark cycles over 7 days. Data show the mean ± SD of 16 animals in 4 cages on 7 consecutive days. Additionally, corresponding statistical data are listed (**p < 0.01, ***p < 0.001, n.s. = not significant).

For preference tests, powdered standard chow (STD) was added to each test food (1:1) to exclude the influence of organoleptic properties (Fig. 1a)^3^. It had been shown before that the order and duration of the test episodes did not influence the outcome^3^. At first, the relative intake increased with increasing fat and, hence, energy content of the test foods with a maximum at a composition of 35% fat and 45% carbohydrates. Higher fat contents, however, led to decreased food intake (Fig. 1a). Because fat has higher energy density than carbohydrates, these findings indicate that the energy content is not the sole determinant of food intake in non-deprived rats. Remarkably, the mean fat/carbohydrate ratio of the most attractive test foods almost exactly matched the composition of potato chips (Fig. 1a). It remains to be investigated if the above conclusion can be extended to other food products with a similar fat/carbohydrate ratio such as chocolate or other snack food.

We have recently shown that the consumption of potato chips in ad libitum fed rats strongly modulates the whole brain activity mainly affecting the reward circuit and systems related to food intake, sleep, and locomotor activity^3^. Therefore, the present study investigated the impact of the fat/carbohydrate ratio of the test food on these modulations. For this purpose, ad libitum fed rats were exposed to a test food containing 35% fat and 65% carbohydrates (FCH) as an almost isocaloric (565 vs. 535 kcal/100 g) model for potato chips. A control group received powdered STD instead. Afterwards, changes in the whole brain activity pattern during the feeding phase were recorded by manganese-enhanced magnetic resonance imaging (MEMRI)^5^ as previously described^3^. According to the study design shown in Fig. 1b, a training phase (TP) offering the test foods ad libitum was followed by an intermediate phase without test food (seven days each). Prior to MEMRI measurement, the contrast agent manganese chloride was administered by dorsally subcutaneously implanted osmotic pumps to map the integrated brain activity during the following seven days. During this manganese phase (MnP), the rats had reinstated access to their already known test food. Standard pellet chow and tap water were available ad libitum throughout the entire study (Fig. 1b). This test setup compared energy intake as well as whole brain activity pattern of both groups and resulted in significantly elevated energy intake in the FCH group during TP and MnP in the light as well as in the dark cycle of the day compared to the control (Fig. 1b). Additionally, locomotor activity of single rats near the food dispensers was counted. In contrast to other locomotor assays, such as the open field test measuring general locomotor activity and anxiety, the feeding-related locomotor activity, which was assessed in the present study, rather reflects food-seeking behaviour. Feeding-related locomotor activity, however, was only slightly elevated when FCH was available instead of powdered STD during the dark cycle of TP (mean locomotor activity [counts] STD 205 ± 46, FCH 230 ± 41, n = 4, p = 0.0633) and MnP (mean locomotor activity [counts] STD 155 ± 24, FCH 164 ± 17, n = 4, p = 0.2123) (Fig. 1b). In contrast, the access to potato chips led to a much higher feeding-related locomotor activity compared to the same STD control group during the dark cycle^3^, which was significant both in TP (mean locomotor activity [counts] STD 205 ± 46, potato chips 290 ± 52, n = 4, p < 0.001) and in MnP (mean locomotor activity [counts] STD 155 ± 24, potato chips 197 ± 29, n = 4, p = 0.0011). Thus, it can be concluded that the fat/carbohydrate ratio determines the palatability of potato chips, but that the feeding behaviour is also influenced by other components in snack food. It remains speculative, however, if these differences relate to “wanting”- and “liking” aspects of food intake^6^.

**Figure 2:**
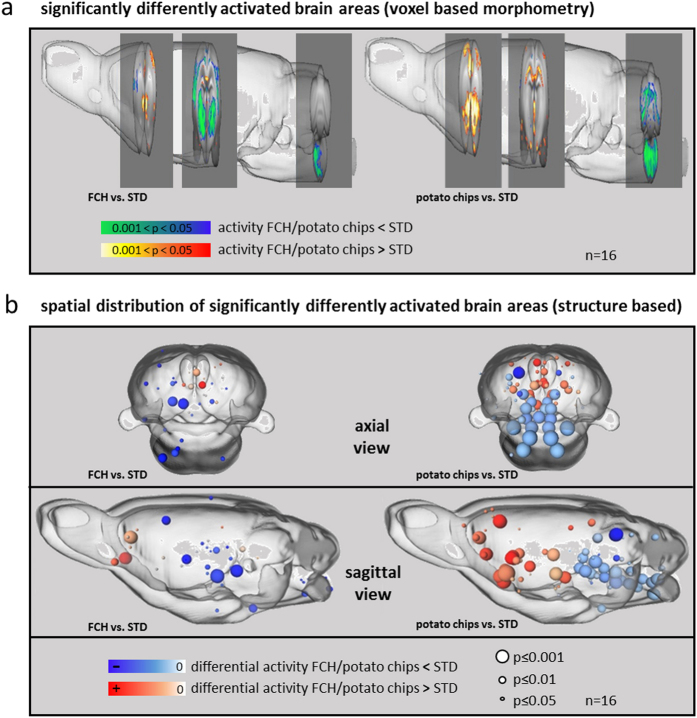
(a) Significantly differently activated brain areas (mixture of 35% fat/ 65% carbohydrate (FCH) vs. standard chow (STD) and potato chips vs. STD^3^) by a voxel-based morphometric analysis exemplified for three slices displayed in the average rat brain surface. Mean data of the food group fat/carbohydrate (FCH, left column) are compared to changes in brain activity pattern induced by potato chips under the same conditions (reviewed from Hoch *et al.* 2013^3^, right column). **(b) 3D distribution of significantly differently activated brain areas displayed in axial and sagittal view** (35% fat/ 65% carbohydrate test food FCH vs. STD, left column, and potato chips vs. STD, right column, reviewed from Hoch *et al.* 2013^3^). Blue spheres symbolize brain areas with lower, red spheres brain regions with higher activity after the intake of the respective test food FCH or potato chips^3^, each compared to STD. The size of the spheres symbolizes significance levels (small: p ≤ 0.05, medium: p ≤ 0.01, big: p ≤ 0.001, n = 16).

**Figure 3:**
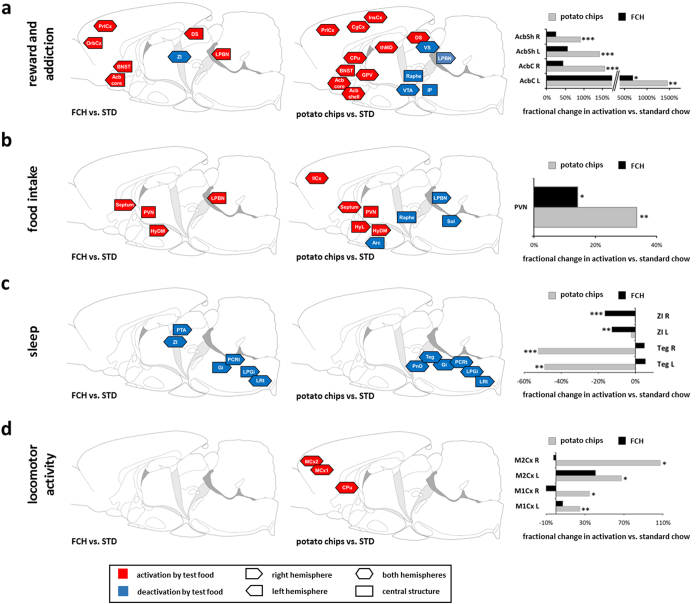
Brain regions assigned to the functional groups (a) “reward and addiction“, (b) “food intake”, (c) “sleep”, and (d) “locomotor activity” on a schematic sagittal view of the rat brain with significantly different (p < 0.05) manganese accumulation in brain structures of ad libitum fed rats with additional access to 35% fat/ 65% carbohydrate test food (FCH, first column) or the snack food potato chips (reviewed from Hoch *et al*. 2013^3^, second column). Red rectangles symbolize brain regions significantly activated by the snack food potato chips or FCH, both vs. powdered standard chow (STD), blue rectangles respective brain regions with higher activity due to the intake of powdered STD vs. snack food potato chips or FCH. Triangles attached to the rectangles left and/or right indicate the hemisphere of significant differences. Rectangles without triangles represent central brain structures. The third column shows the fractional change of snack food and FCH, respectively, vs. STD (***p < 0.001, **p < 0.01, *p < 0.05, n = 16). Acb core: core region of the nucleus accumbens; Acb shell: shell region of the nucleus accumbens, Arc: arcuate hypothalamic nucleus, BNST: bed nucleus of stria terminalis, CgCx: cingulate cortex, CPu: caudate putamen (stratium), DS: dorsal subiculum, Gi: gigantocellular nucleus, GPV: ventral pallidum, HyDM: dorsomedial hypothalamus, HyL: lateral hypothalamus, IlCx: infralimbic cortex, InsCx: insular cortex, IP: interpeduncular nucleus, LPBN: lateral parabrachial nucleus, LPGi: lateral paragigantocellular nucleus, LRt: lateral reticular nucleus, MCx1: primary motor cortex, MCx2: secondary motor cortex, OrbCx: orbital cortex, PCRt: parvicellular reticular nucleus, PnO: pontine reticular nucleus oral, PrlCx: prelimbic cortex, PTA: pretectal area, PVN: paraventricular thalamic nucleus anterior, Raphe: raphe nucleus, Septum: septum, Sol: solitary tract, Teg: tegmental nuclei, thMD: mediodorsal thalamic, VS: ventral subiculum, VTA: ventral tegmental area, ZI: zona incerta.

Whole brain activity monitoring by MEMRI revealed significant differences in the activation of brain areas by the intake of FCH compared to STD (Fig. 2a,b, Fig. 3, first column, Table 1). The present results were compared with previous MEMRI analyses of the modulation of brain activity pattern during the intake of potato chips vs. STD under the very same conditions^3^. The former data are listed in the second column of Figs. 2 and 3. Although FCH had a similar fat/carbohydrate ratio and nearly identical energy density compared to potato chips, FCH activated a much smaller number (33) of brain areas significantly differently from STD than potato chips (78 areas, Fig. 2). Effects were detected in the functional groups related to reward and addiction (Fig. 3a), food intake (Fig. 3b), sleep (Fig. 3c), and locomotor activity (Fig. 3d). Figure 2b shows an overview of all significantly differently activated brain areas comparing the effects of FCH and potato chips, respectively, with those of STD. Additionally, the fractional change in activation, i.e. the manganese uptake reflecting neuronal activity, differs decisively regarding the consumption of FCH vs. STD compared to potato chips vs. STD (Fig. 3, third column). The nucleus accumbens is considered to be a main structure of the reward system^7^. The consumption of FCH led to a significantly 7.8-fold increased activation in one of the four substructures, the core subregion of the left hemisphere. The increase in the shell subregions as well as in the core subregion of the right hemisphere was not significant (Fig. 3a). The intake of potato chips under similar conditions also led to the highest activation by far of the left core subregion of the nucleus accumbens. Compared to FCH, however, the activation level in this substructure was even twofold higher. In contrast to FCH, the three other substructures were also significantly activated compared to the control (Fig. 3a). Thus, it can be concluded that FCH activates reward systems in the brain, but with minor effect than potato chips. This conclusion is also reflected by other structures of the reward/addiction system, which were significantly activated by the intake of potato chips and FCH, such as the bed nucleus of stria terminalis (left hemisphere)^8^, the dorsal subiculum^9^, or the prelimbic cortex (right and left hemisphere)^0^. Other brain structures, in contrast, were not significantly affected by the intake of FCH, even though they are important components of the reward circuits and were clearly modulated by the intake of potato chips, such as the ventral pallidum, the ventral tegmental area, or the caudate putamen (Table 1)^3^.

Similar conclusions can be drawn from the analysis of brain circuits that are associated with food intake. For example, the dorsomedial hypothalamus, the septum as well as the paraventricular thalamic nucleus, which were activated during the intake of FCH and potato chips, can be linked to the control of food intake^2^. But again, FCH failed to modulate other structures of the satiety circuits, which were deactivated by potato chips, such as the arcuate hypothalamic nucleus or the solitary tract. In addition, the intensity of activation was lower by FCH than by potato chips, which was reflected, for example, by a 2.3-fold significantly higher activation of the paraventricular thalamic nucleus anterior (Fig. 3b). These data suggest that FCH modulates brain structures related to food intake differently from STD, an effect which may be reflected by the higher energy intake through FCH (Fig. 1b).

The intake of FCH also led to a strong deactivation of brain structures linked to sleep. Some brain areas were only deactivated by FCH such as the zona incerta (Fig. 3c), whereas other areas were only deactivated by potato chips, such as the tegmental nuclei. Although eight sleep-related structures were modulated by FCH and eleven by potato chips, the effect of both test foods seems to be in a similar range. Because this result was not expected, the duration of sleep was not measured in the present study so that it is not clear, if the FCH-induced modulation of sleep circuits correlates with a modulation of sleeping behaviour.

Brain regions responsible for locomotor activity and movement in general were not significantly influenced by the intake of FCH compared to STD (Fig. 3d, first column). This is concurrent with the behavioural observations that FCH induced only a slightly, but non-significantly higher food-related locomotor activity compared to STD (Fig. 1b). In contrast, it was shown that the activation of the structures of the motor system in the brains of rats with access to potato chips was accompanied by an elevated feeding-related locomotor activity^3^.

It is not fully clear if the observed activation pattern is related to hedonic hyperphagia. In contrast to homeostatic food intake, which is controlled by the energy level of the organism, hedonic food intake is mediated by the reward generated by some foods^3^. Since hedonic food intake is not heavily linked to the energy needs, it often leads to hyperphagia. Models have been developed that describe the neural correlates of hedonic hyperphagia. Berthoud, for example, suggests that homeostatic food intake is linked to leptin-sensitive circuits that include mainly the arcuate nucleus and the nucleus of the solitary tract, but also involve a wide range of other areas including hypothalamic sites, such as the paraventricular nucleus or the nucleus accumbens^4^. This homeostatic regulation of food intake may, however, be overruled by reward signals such as components of liking and wanting^5^. Liking of food was related to mu-opioid signalling in the nucleus accumbens, ventral pallidum, parabrachial nucleus and the nucleus of the solitary tract^4^, whereas wanting of food was related to the dopamine system in the ventral tegmental area, nucleus accumbens, prefrontal cortex, amygdala, and hypothalamus. Kenny additionally emphasized the contribution of the insular cortex, which is supposed to store information on the hedonic properties of food and may also be linked to craving^0^. In contrast to the brain activation pattern linked to potato chip intake, only few of these areas associated with hedonic hyperphagia were actually influenced by the intake of FCH. Therefore, extended behavioural experiments are required to investigate if the preference of FCH is actually accompanied by hyperphagia.

To date, it is not clear which molecular components of potato chips are responsible for the stronger brain modulation effects of this test food. Since a salted, but unseasoned product without flavour enhancer addition was used, salt, flavour, and minor amounts of proteins were present besides the main components fat and carbohydrates. Furthermore, molecular changes that occur during processing have to be considered. It was shown before that the taste of salt induced Fos expression in the nucleus accumbens of salt-deprived rats. The intake of salt in non-depleted animals, in contrast, did not lead to an activation of this structure of the reward system^6^. Moreover, it has been reported that the intake of salt in solid food rather produced an aversive effect in rats^7^. Therefore, it does not seem likely that salt was a main modulator of the brain reward system in the present experiments. The previously introduced two-choice preference test may now serve to further investigate the influence of other potato chips components on food intake.

We conclude from our behavioural data that the ratio of fat and carbohydrates, but not the absolute energy density, is the major determinant of the palatability and intake of snack food during short-term two-choice preference tests in rats. Moreover, the intake of the FCH mixture, which is nearly isocaloric to potato chips, induced the maximum energy intake in ad libitum fed rats, which was accompanied with significantly different activation of brain structures related to reward, food intake, and sleep. The intake of potato chips under the same conditions led to a much larger number of differently activated brain structures in these circuits and also to a clearly higher fractional change compared to STD. Thus, from the imaging approach, it can be concluded that the energy density alone is only a moderate determinant of the rewarding properties of snack food. Although the ratio of fat and carbohydrates of potato chips seems to be highly attractive, it can be hypothesized that other molecular determinants exist in this snack food, which modulate the activity of brain circuits, particularly the reward system, even stronger and lead to increased food seeking behaviour.

## Methods

### Ethics statement

This study was carried out in strict accordance with the recommendations of the Guide for the Care and Use of Laboratory Animals of the National Institutes of Health. The protocol was approved by the Committee on the Ethics of Animal Experiments of the Friedrich-Alexander Universität Erlangen-Nürnberg (Regierung Mittelfranken, Permit Number: 54-2532.1-28/12).

### Preference test

Preference tests were conducted as previously described three times a day during the light cycle for 10 minutes each with 20–36 repetitions in total per test food against the reference^3^. This test schedule provides sufficient data points for the evaluation of a food preference. The tests were conducted with 8 male Wistar rats (2 cages with 4 animals each, 571 ± 41 g, purchased from Charles River, Sulzfeld, Germany) and reproduced with 10 male Sprague Dawley rats (2 cages with 5 animals each, initial weight 543 ± 71 g, purchased from Charles River, Sulzfeld, Germany), which had been trained for the test. Thus, the number of animals which performed each test was 18 and the number of cages 4 (four biological replicates). Each experiment was repeated 5–6 times with each animal group. All rats were kept in a 12/12 h dark/light cycle. The rats had access to standard chow pellets (Altromin 1324, Lage, Germany, 4 g/100 g fat (F), 52.5 g/100 g carbohydrates (CH), 19 g/100 g protein (P)) in addition to the test foods and to tap water ad libitum throughout the whole study. Test foods with different ratios of F (sunflower oil, purchased from a local supermarket) and CH (maltodextrin, dextrin 15 from maize starch, Fluka, Germany), mixed with 50% powdered STD were used to compare the respective activity to induce food intake. Powdered STD was added to minimize textural and sensory influences on the consumption. As reference food for all behavioural preference tests, a mixture of 50% powdered STD, 17.5% F, and 32.5% CH was used, which has a highly similar F/CH composition as 50% potato chips in STD and has been used as a model for 50% potato chips in STD before^3^. Additionally, we tested foods composed of 50% powdered STD with the additions of the following mixtures of F and CH (%F/%CH): 5/45, 10/40, 17.5/32.5, 25/25, 30/20, 35/15, 40/10, 45/5, and 50/0. Considering the composition of 50% STD, the reference food contained in total (%F/%CH) 20/59, the other test foods 7/71, 12/66, 20/59, 27/51, 32/46, 37/41, 42/36, 47/31, and 52/26. The contents of all other components of powdered STD like protein (9%), fiber (3%), or minerals (ash, 3.5%) were constant in all test foods.

The energy intake dependent on the respective test food was calculated by multiplication of the ingested amount of the test food with its respective energy content. The relative contribution of one test food to the sum of ingested test food and reference was calculated by dividing the amount of the respective test food by the total intake of test food and reference.

### Recording of the behavioural data for energy intake and feeding-related locomotor activity

Behavioural data were recorded as previously described^3^. Briefly, test food intake was measured on a daily basis and energy intake was calculated by multiplication of the mass of the ingested test food with the respective energy content. Feeding-related locomotor activity was quantified via webcam pictures which were taken every 10 seconds from above the cage. One count was defined as “one rat shows locomotor activity near one food dispenser”. For statistical evaluation Student’s t-tests (two-tailed) were performed using the mean value (energy intake or feeding-related locomotor activity) during 7 days (TP or MnP) per cage (n = 4 cages, with 16 rats in total in each group).

### Recording of whole brain activity pattern by MEMRI

Male Wistar rats (initial weight 261 ± 19 g, purchased from Charles River, Sulzfeld, Germany) kept in a 12/12 h dark/light cycle were randomly divided into two groups. Both groups had ad libitum access to standard chow pellets (Altromin 1324, Altromin, Lage, Germany) over the whole course of the study.

One group (n = 16, initial body weight 256 ± 21 g) received powdered STD (Altromin 1321) and the other group (n = 16, initial body weight 266 ± 16 g) received a mixture of 35% F (sunflower oil, purchased from a local supermarket) and 65% CH (maltodextrin, dextrin 15 from maize starch, Fluka, Taufkirchen, Germany) additionally to the standard chow pellets. The present study was run in parallel to the previously published study on potato chips^3^, so that the same control group could be used allowing maximum comparability of the data sets.

MEMRI (on a 4.7 T Bruker MRI using an optimized modified driven equilibrium Fourier transform (MDEFT) sequence) was used to map the brain activation with a fine resolution of 109 × 109 × 440 μm (for details see Hoch *et al.* 2013^3^). Because the sensitivity of MEMRI is lower compared to the preference tests, the test foods were presented for a longer period of time. Recordings require relative high concentrations of the potentially toxic contrast agent manganese, which reaches the brain only several hours after application. To avoid negative side effects on basic physiology and behaviour of the animals due to the injection of the manganese chloride solution in doses sufficient for MEMRI measurement, osmotic pumps served for the gentle, but rather time-consuming continuous application of non-toxic amounts of manganese, which accumulated in the activated brain areas during the whole time course of the 7-day food test phase^8^. Study design, preparation of the osmotic pumps, parameters for MRI measurements, data processing as well as the recording of food intake and feeding-related locomotor activity has been previously described^3^. The original MRI grey values of the segmented brain per animal were registered by a non-rigid registration workflow^3^. Based on these registered datasets a voxel-based morphometric analysis was performed and the resulting statistical parameters were visualized. Z-Score based Student’s t-tests were performed to detect significant differences in brain activation. For 3D visualization of the distribution of the significantly differently activated brain structures, we represented each brain structure as a sphere at its centre of gravity. The coordinates were derived from a 3D digital brain atlas. The radius of each sphere was used to code its significance level and the intensity shading codes the activity difference to STD.

## Author Contributions

Conceived and designed the experiments: T.H. M.P. A.H. Performed the experiments: T.H. A.H. Analysed the data: T.H. S.K. S.G. A.H. Interpreted the data T.H. M.P. A.H. Contributed reagents/materials/analysis tools: A.H. M.P. Wrote the paper: T.H. M.P. A.H.

## Additional Information

**How to cite this article**: Hoch, T. *et al.* Fat/carbohydrate ratio but not energy density determines snack food intake and activates brain reward areas. *Sci. Rep.* **5**, 10041; doi: 10.1038/srep10041 (2015).

## Figures and Tables

**Table 1:** Z-Scores of significantly differently activated brain areas comparing rats with access either to standard chow only or to a mixture of fat and carbohydrates and the respective p-values of t-statistics, n = 16.

**Brain structure**	**Z-Score ± SD**		**p-Value**
	**Standard chow**	**Fat & carbohydrates**	
Reward and addiction			
Bed nucleus of stria terminalis L^8^	0.68 ± 0.38	0.88 ± 0.28	0.0249
Dorsal subiculum L^9^	−0.98 ± 0.53	−0.48 ± 0.98	0.0178
Lateral parabrachial nucleus L^9^	−0.07 ± 0.46	0.25 ± 0.68	0.0329
Nucleus accumbens (core subregion) L^0^	0.03 ± 0.30	0.21 ± 0.36	0.0386
Orbital cortex L^0^	−0.41 ± 0.60	−0.03 ± 0.77	0.0388
Prelimbic cortex L^0^	−1.11 ± 0.27	−0.71 ± 0.56	0.0010
Prelimbic cortex R^7^	−1.06 ± 0.41	−0.80 ± 0.51	0.0356
Zona incerta L^1^	1.56 ± 0.25	1.36 ± 0.31	0.0087
Zona incerta R^1^	1.24 ± 0.19	1.03 ± 0.19	0.0001

Food Intake			
Dorsomedial hypothalamus R^1^	−2.00 ± 0.31	−1.81 ± 0.37	0.0387
Lateral parabrachial nucleus L^9^	−0.07 ± 0.46	0.25 ± 0.68	0.0329
Paraventricular thalamic nucleus anterior^2^	−1.04 ± 0.19	-0.89 ± 0.25	0.0118
Septum R^2^	−0.21 ± 0.44	0.05 ± 0.47	0.0318

Sleep			
Gigantocellular nucleus R^3^	−0.36 ± 0.32	−0.55 ± 0.32	0.0219
Lateral paragigantocellular nucleus R^4^	−0.97 ± 0.27	−1.15 ± 0.26	0.0100
Lateral reticular nucleus L^5^	−0.92 ± 0.35	−1.12 ± 0.39	0.0378
Lateral reticular nucleus R^5^	−1.03 ± 0.35	−1.28 ± 0.35	0.0072
Parvicellular reticular nucleus R^5^	−0.29 ± 0.29	−0.47 ± 0.25	0.0148
Pretectal area R^6^	2.10 ± 0.43	1.84 ± 0.55	0.0402
Zona incerta L^7^	1.56 ± 0.25	1.36 ± 0.31	0.0087
Zona incerta R^7^	1.24 ± 0.19	1.03 ± 0.19	0.0001
